# The Oxford Elbow Score demonstrated good measurement properties when used with a shortened 7-day recall period

**DOI:** 10.1016/j.jseint.2022.12.023

**Published:** 2023-01-31

**Authors:** Eythor Ö. Jonsson, Johan Wänström, Hanna Björnsson Hallgren, Lars Adolfsson

**Affiliations:** a Department of Orthopaedics, Institute of Clinical Sciences, The Sahlgrenska Academy, University of Gothenburg, Gothenburg, Sweden; b Department of Orthopaedics, Sahlgrenska University Hospital, Mölndal, Sweden; c Division of Orthopaedic Surgery, Department of Biomedical and Clinical Sciences, Linkoping University, Linkoping, Sweden

**Keywords:** Recall period, Oxford elbow score, Validation, Measurement properties, Elbow, Patient-reported outcome measures

## Abstract

**Background:**

The Oxford Elbow Score (OES) is a well-validated, elbow-specific, patient-reported outcome measure (PROM), originally assigned a 4-week recall period. For PROMs, short recall periods could have some advantages, such as optimizing validity by minimizing the negative effects of inaccurate recollection and temporal trends (increase or decrease) in symptoms over the course of the recall period. Temporal trends in elbow function can, for example, be expected to occur over 4 weeks in patients recovering from an injury or surgery. The purpose of this study was to evaluate the measurement properties of the OES using a shortened, 7-day, recall period (OES-7d).

**Methods:**

The inclusion criteria were fracture, tendon rupture or dislocation affecting the elbow, and age ≥18 years. Patients with Quick Disabilities of the Arm, Shoulder and Hand (QuickDASH) scores of ≥10 points preinjury (pre-existing upper extremity condition) or concurrent upper extremity injuries were excluded. Patients completed the OES-7d, QuickDASH, and Single Assessment Numeric Evaluation-Function for the last 7 days preinjury (T1), the first 7 days postinjury (T2) and a 7-day period 3-5 months postinjury (T3). Correlations were assessed with Spearman’s rho. Analyses of construct validity (correlation between scores) and internal consistency (Cronbach’s alpha) were based on T3 data. Responsiveness was assessed by correlating changes in scores (change scores) between time points. Intra-rater reliability was assessed by calculating intraclass correlation coefficients based on 2 administrations (1- to 3-week interval) of PROMs in a separate group of patients who had sustained an elbow injury 1-2 years previously.

**Results:**

Seventy-five patients (45 women) were included between May 2020 and July 2021. Their mean age was 51.7 years. At T3, Spearman’s rho was −0.91 for the correlation between OES total and QuickDASH scores and 0.76 for the correlation between OES total scores and Single Assessment Numeric Evaluation-Function values (construct validity). Spearman’s rho for correlation between OES total and QuickDASH change scores from T2 to T3 (T3 minus T2) was −0.85 (responsiveness for improvement) and −0.88 for change scores from T1 to T2 (T2 minus T1, responsiveness for deterioration). For the OES domains, Cronbach’s alpha was 0.83 for elbow function, 0.91 for pain and 0.90 for social-psychological domains. The intraclass correlation coefficient for the OES total score was 0.96.

**Conclusion:**

The OES demonstrated good measurement properties when used with a 7-day recall period (OES-7d). These results further establish the OES as a well-validated, elbow-specific PROM and support using a 7-day recall period.

Patient-reported outcomes (PROs) are becoming increasingly recognized as an important outcome parameter in the field of orthopedics.^13^^,^^21^ A commonly cited definition used by the United States Food and Drug Administration is that a PRO is: “any report of the status of a patient’s health condition that comes directly from the patient, without interpretation of the patient’s response by a clinician or anyone else”.^48^ For a patient-reported outcome measure (PROM) to be useful, it needs to have acceptable validity, reliability and responsiveness, characteristics referred to as measurement properties.^24^

PROMs are being used increasingly in elbow literature.^10^ There is, however, a lack of well-validated PROMs for elbow conditions. Using the COnsensus-based Standards for the selection of health Measurement INstruments (COSMIN) checklist, The et al found that the Oxford Elbow Score (OES) was the only elbow-specific rating system for which measurement properties have been assessed with high-quality methodology.^44^ The OES demonstrated good measurement properties when it was initially described by Dawson et al in 2008.^8^ Several studies, also using the original 4-week recall period, have since reported favorable measurement properties for the OES.^9^^,^^18^^,^^27^^,^^29^^,^^33^

The results of PROMs can be affected by the length of the recall period associated with the instrument.^42^ A recall period is a specified time interval on which respondents to PROMs are requested to base their answers to items. The most appropriate length of a recall period varies,^37^ based on factors such as the nature of the condition under study (eg, variability, frequency, and intensity of symptoms), the concept the PROM aims to measure, the ability of the patient reliably to recall information over the period, and the design and length of the study.^28^ As a result, the most suitable length of the recall period for a given PROM can differ, depending on the circumstances. For example, the accuracy of assessing symptoms by recall can be affected by an increase or decrease in symptoms (temporal trends) over the course of a recall period.^40^ Recall periods that are shorter than the 4 weeks initially assigned to the OES might therefore be preferable for assessments when temporal trends in function can be expected, such as while patients are recovering from injury or surgery. Other potential advantages of short recall periods for PROMs are that the results might be less influenced by inaccurate recollection,^31^^,^^48^ assessments can be made more frequently without any overlap of recall periods^28^ and can be made sooner after events such as surgery without truncating the recall period.^42^ In addition, previous studies suggest that shortened recall periods might improve the responsiveness of PROMs.^6^^,^^19^

The purpose of this study was to evaluate the measurement properties of the OES using a shortened 7-day recall period (OES-7d). The OES-7d was hypothesized to have good measurement properties.

## Materials and methods

### Study design and settings

This is a cohort study based on prospectively collected data. Patients were recruited at 3 hospitals in Sweden: Linköping University Hospital, Linköping, Sahlgrenska University Hospital, Gothenburg, and Helsingborg Hospital, Helsingborg. All patients provided written informed consent to participate in the study.

### Eligibility criteria

The inclusion criteria were fracture, tendon rupture or dislocation affecting the elbow and age ≥18 years. Patients treated with and without surgery were included. The exclusion criteria were pre-existing upper extremity condition defined as a Quick Disabilities of the Arm, Shoulder and Hand (QuickDASH) score of >10 points for the last 7 days prior to injury (see below), inability to participate in assessments (eg, cognitive impairment or insufficient understanding of the Swedish language) or concurrent upper extremity injury.

### Outcome measures

The OES is a PROM developed to assess the outcome after surgery on the elbow.^8^ The OES consists of 12 items grouped into 3 domains: elbow function, pain, and social-psychological domains. Each item has 5 response options that are scored from 0 to 4, with lower numbers representing greater impairment. The results are summarized by dividing the total score for the items by the maximum possible score and then multiplying it by 100. In this study, patients were requested to respond to items based on their experiences during the past 7 days (OES-7d) as opposed to 4 weeks in the original version. The header of each item in the OES was changed accordingly, while the item itself was left unchanged.

The QuickDASH^4^ is an 11-item shortened version of the Disabilities of the Arm, Shoulder, and Hand (DASH)^17^ which is intended to assess physical function in populations with upper extremity musculoskeletal conditions. The QuickDASH is used with a 1-week recall period and scored from 0 to 100, with higher scores representing poorer function. A Swedish language version of the QuickDASH, previously found to be a valid outcome measure for upper extremity disorders, was used.^15^

In this study, a modified version of the Single Assessment Numeric Evaluation (SANE)^49^ was used, referred to as SANE-Function (SANE-F). The SANE was described by Williams et al in 1999 as the following question: “How would you rate your shoulder today as a percentage of normal (0%-100% scale with 100% being normal)?”.^49^ The instrument has since been adapted for other anatomical regions including the elbow.^30^^,^^36^ SANE is often referred to as assessing function.^30^^,^^39^^,^^45^^,^^50^ Consequently, some studies have used and assessed the construct validity of a question modified by incorporating the term “function”.^14^^,^^32^ The following question was used in the current study: How would you rate your current elbow function compared with a completely normal elbow? Responses were recorded on an 11-step numerical rating scale.^2^^,^^46^

### Data collection

Patients responded to the OES, QuickDASH, and SANE-F for 3 different periods of time: the last 7 days prior to injury (T1), the first 7 days after injury (T2), and a period of 7 days 3-5 months after injury (T3). T1 questionnaires, answered by recall, were completed within 14 days from injury, and T2 questionnaires were completed within 7-21 days from injury. PROMs were completed at home and returned by mail or at the hospital, in connection with either surgical treatment or visits to the outpatient department.

### Translation of the OES

A Swedish language version of the OES was not previously available. A translation process was therefore set up according to the guidelines from Beaton et al.^3^ The OES was translated into Swedish and then back into English (back-translation) by 2 professional translators whose native language is English. The back translation was then compared with the original version by a professional translator not involved in prior steps in the process and any discrepancies were resolved by a consensus discussion between the groups of translators. Permission for the use of the OES and approval of the translation process was obtained from Oxford University Innovation Ltd.

### Data management and statistical analysis

The data were computerized with Filemaker Pro, version 15 (Claris International, Santa Clara, CA, USA). QuickDASH questionnaires with 1 or more missing items were excluded.^4^ OES questionnaires with any missing item were excluded. The data were analyzed in SPSS, version 26 (IBM Corp., Armonk, NY, USA). Continuous variables are presented as mean and standard deviation (SD), while categorical variables are presented as number (n) and percent (%). Comparisons of means between time points were made with paired samples t-tests. The results of statistical tests were considered significant when the *P* value was less than .05. Measurement properties were assessed with reference to the COSMIN checklist^25^ and the COSMIN terminology was adhered to.^24^

Construct validity was assessed by calculating Spearman’s correlation coefficients (Spearman’s rho, *r*_s_) between scores for the OES, QuickDASH, and SANE-F at T3. One way of arbitrarily grading the degree of correlations is negligible, *r*_s_ < 0.3; low, 0.3 ≤ *r*_s_ < 0.5; moderate, 0.5 ≤ *r*_s_ < 0.7; high, 0.7 ≤ *r*_s_ < 0.9 and very high, 0.9 ≤ *r*_s_ ≤ 1.^26^ Based on previous work, the hypothesis was that, at T3, Spearman’s rho would be at least −0.75 for the correlation between QuickDASH and OES total scores and at least −0.6 between scores for the QuickDASH and the OES domains (construct validity).^18^ It was hypothesized that the value of *r*_s_ was at least 0.5 for the correlation between OES scores and SANE-F values, as these instruments were assumed to measure similar constructs.^34^

To assess responsiveness, differences in scores between time points were calculated (change scores). Change scores between T1 and T2 (T2 minus T1) and T2 and T3 (T3 minus T2) are referred to as T1/T2 and T2/T3, respectively. Responsiveness, also known as longitudinal validity,^43^ was assessed by calculating *r*_s_ for correlations between change scores for the OES, QuickDASH, and SANE-F for both T1/T2 (improvement) and T2/T3 (deterioration). All 3 instruments were assumed to measure similar constructs and a generic hypothesis of absolute values of *r*_s_ ≥ 0.5 was therefore formulated for correlations between change scores.^34^ In the case of correlations between OES and QuickDASH change scores T2/T3, this level also seemed reasonable based on previous studies.^7^^,^^18^ The internal consistency of the domains of the OES was assessed with Cronbach’s alpha based on data at T3.

### Intrarater reliability

Intrarater reliability was assessed in a separate group of patients, who had suffered an elbow injury 1-2 years prior to assessment. Patients were excluded if, since the time of injury, they had incurred a new upper extremity injury, undergone surgery on the upper extremities, or experienced an exacerbation of an upper extremity condition. Patients received questionnaires by mail, completing the OES, QuickDASH, and SANE-F on two occasions, with a 1- to 3-week interval. Intraclass correlation coefficients (ICCs) were calculated using a single-measurement, absolute agreement, 2-way mixed-effects model.^35^

## Results

### Demographic-, injury- and treatment-related variables

Between May 2020 and July 2021, 75 patients were included. Their overall mean age was 51.7 years (17.8) and there were 45 women. Injury-related characteristics are presented in Table I. Radial head fracture (n = 32) was the most common type of injury, Table II. Fifty-four patients were treated with surgery. Open reduction and internal fixation was the most common surgical procedure, Table II. The mean number of days that passed between the injury and surgery was 5.7 (3.9).

**Table I:** Injury-related characteristics.

	n (%)
Injured side	
Right	31 (41)
Left	44 (59)
Dominant side injured	29 (39)
Injury mechanism	
Ground level fall	40 (53)
Fall from height	9 (12)
Bicycle accident	10 (13)
Sports	6 (8)
Other	10 (13)
Low-energy injury mechanism	69 (92)

**Table II:** The distribution of types of injury and their treatment.

Type of injury	Nonoperative treatment (n = 21)	Operative treatment (n = 54)			
		ORIF∗ (n = 29)	Radial head arthroplasty∗ (n = 13)	Ligament repair (n = 5)	Other (n = 7)
Type of fracture					
Radial head	14	9	8		1†
Distal humerus		5			2‡
Monteggia		3			3§
Olecranon		5			
Radial neck	5				
Coronoid	1	2			
Terrible triad injury		5	5		
Elbow dislocation				5	
Distal biceps rupture	1				1‖

*ORIF*, open reduction and internal fixation.

∗ And repair of associated ligament injuries, anterior capsule, and coronoid fragments when appropriate.

† Radial head resection and ligament repair.

‡ Elbow hemiarthroplasty.

§ ORIF, of ulnar fracture and radial head arthroplasty.

‖ Tendon reinsertion.

### Results of the OES, QuickDASH, and SANE-F

There were no missing values for the OES. The number of patients with 1 missing value for the QuickDASH for each of the 3 periods of time was 1 for T1, 6 for T2, and 4 for T3. For the 3 time periods, the mean number of days between injury and completing questionnaires was (SD): T1, 6.2 (3.4); T2, 9.6 (2.7); and T3, 106 (14.7). There was a deterioration between T1 and T2 in terms of mean scores for the OES, QuickDASH, and SANE-F (*P* < .001), Table III. Between T2 and T3, the mean scores for PROMs showed a statistically significant improvement (*P* < .001).

**Table III:** Results of the OES, QuickDASH, and SANE-F at T1, T2, and T3.

	T1∗	T2†	T3‡
	Mean (SD) n	Mean (SD) n	Mean (SD) n
OES			
Total score	99.9 (0.8) 74	31.5 (14.7) 35	74.5 (19.1) 75
Elbow function	100 (0) 74	31.8 (14.5) 35	80.0 (18.4) 75
Pain	99.7 (2.3) 74	42.0 (21.4) 35	77.3 (21.2) 75
Social-Psychological	100 (0) 74	20.9 (16.5) 35	66.3 (25.5) 75
QuickDASH	0.9 (2.3) 75	65.3 (16.9) 33	22.0 (19.2) 75
SANE-F	9.9 (0.4) 73	2.5 (1.9) 30	6.9 (2.0) 74

*OES*, Oxford Elbow Score; *DASH*, Disabilities of the Arm, Shoulder and Hand; *SANE-F*, Single Assessment Numeric Evaluation-Function.

∗ T1, the last 7 days before injury.

† T2, the first 7 days after injury.

‡ T3, a period of 7 days after 3-5 months from injury.

### Construct validity

Spearman’s rho for the correlation between OES total and QuickDASH scores at T3 was −0.91, Table IV. At T2 (n = 35), *r*_s_ was −0.86 for the correlation between OES total and QuickDASH scores and 0.31 for the correlation between OES total scores and SANE-F values.

**Table IV:** Assessment of construct validity based on data at T3. Spearman’s correlation coefficients (rs) between the OES (and its domains), Quick-DASH, and SANE-F.

	OES				QuickDASH
	Total score	Elbow function	Pain	Social-psychological	
OES					
Total score	-				
Elbow function	0.88	-			
Pain	0.79	0.62	-		
Social-Psychological	0.93	0.77	0.58	-	
QuickDASH	−0.91	−0.82	−0.73	−0.84	-
SANE-F	0.76	0.75	0.60	0.68	−0.79

*OES*, Oxford Elbow Score; *DASH*; Disabilities of the Arm, Shoulder and Hand; *SANE-F*, Single Assessment Numeric Evaluation-Function.

### Responsiveness (longitudinal validity) for improvement

Spearman’s rho for correlation between OES total and QuickDASH change scores for T2/T3 was −0.85, Table V. The following was the correlation (*r*_s_) between QuickDASH change scores and those of the domains of the OES: −0.62 for elbow function, −0.74 for pain, and −0.64 for social-psychological domains. This primary analysis was based on 35 patients who responded to T2 between 7 and 21 days after injury whereof 8 had surgery during the first 7 days following injury, 12 had surgery more than 7 days after injury, and 15 were treated nonsurgically. In order to obtain a larger cohort (n = 57), a secondary analysis group was created by adding 22 patients who responded to T2 between 4 and 6 days after injury to the primary analysis. In the secondary analysis, correlations (*r*_s_) between OES and QuickDASH change scores were −0.81 and 0.54 between OES total and SANE-F change scores.

**Table V:** Responsiveness (longitudinal validity) for improvement assessed with r_s_ between T2/T3 change scores of the OES, QuickDASH, and SANE-F.

	OES				
	Total score	Elbow function	Pain	Social-psychological	QuickDASH
OES					
Total score	-				
Elbow function	0.76	-			
Pain	0.80	0.42	-		
Social-Psychological	0.83	0.60	0.45	-	
QuickDASH	−0.85	−0.62	−0.74	−0.64	-
SANE-F	0.52	0.43	0.37	0.59	−0.42

*r*_*s*_, Spearman's correlation coefficient; *OES*, Oxford Elbow Score, *DASH*; Disabilities of the Arm, Shoulder and Hand; *SANE-F*, Single Assessment Numeric Evaluation-Function.

### Responsiveness (longitudinal validity) for deterioration

Spearman’s rho for the correlation between OES total and QuickDASH change scores for T1/T2 was −0.88, Table VI. The corresponding correlation coefficients (*r*_s_) between change scores for the QuickDASH and the domains of the OES were −0.71 for elbow function, −0.78 for pain, and −0.87 for social-psychological domains. A secondary analysis was performed based on the same group as described for responsiveness for improvement. The correlation (*r*_s_) between OES total and QuickDASH change scores was −0.83, while it was 0.50 between OES total and SANE-F change scores.

**Table VI:** Responsiveness (longitudinal validity) for deterioration assessed with r_s_ between T1/T2 change scores of the OES, QuickDASH and SANE-F.

	OES				
	Total score	Elbow function	Pain	Social-psychological	QuickDASH
OES					
Total score	-				
Elbow function	0.83	-			
Pain	0.90	0.61	-		
Social-Psychological	0.83	0.63	0.62	-	
QuickDASH	−0.88	−0.71	−0.78	−0.87	-
SANE-F	0.38	0.45	0.20	0.46	−0.27

*r*_*s*_, Spearman's correlation coefficient; *OES*, Oxford Elbow Score, *DASH*; Disabilities of the Arm, Shoulder and Hand; *SANE-F*, Single Assessment Numeric Evaluation-Function.

### Internal consistency

The internal consistency (Cronbach’s alpha) of the domains of the OES was 0.83 for elbow function, 0.91 for pain, and 0.90 for social-psychological domain. For none of the items did deletion result in a higher Cronbach’s alpha value for the respective domain.

### Intrarater (test-retest) reliability

Intrarater reliability was assessed in a separate group of 56 patients who had sustained an elbow injury between September 2019 and October 2020. The distribution of diagnoses was the following: distal humeral fracture (n = 15), olecranon fracture (n = 13), terrible triad injury (n = 9), radial head fracture (n = 10), and miscellaneous (n = 9). Forty-five patients had been treated with surgery. The surgical procedures were open reduction and internal fixation (n = 28), radial head arthroplasty (n = 8), elbow hemiarthroplasty (n = 7), and total elbow arthroplasty (n = 2). The mean number of years (SD) from the injury to the first administration of PROMs was 1.2 (0.2). The mean age at the time the questionnaires were completed was 58.7 years (17.8). The mean duration of time between the administrations was 9.1 days (3.2). The ICC for the OES total score was 0.96, Table VII.

**Table VII:** Assessment of intra-rater reliability. ICCs for the OES, QuickDASH, and SANE-F. Based on a group of 56 patients with a history of elbow injury (1-2 yr previously) who completed the questionnaires twice, with 9.1 d between on average.

	ICC
OES	
Total score	0.96
Elbow function	0.91
Pain	0.89
Social-Psychological	0.94
QuickDASH	0.95
SANE-F	0.87

*ICC*, intraclass correlation coefficient; *OES*, Oxford Elbow Score; *DASH*, Disabilities of the Arm, Shoulder and Hand; *SANE-F*, Single Assessment Numeric Evaluation-Function.

## Discussion

The main finding in this study is that the OES demonstrated good measurement properties when used with a shortened 7-day recall period (OES-7d). The measurement properties observed in this study compare well with those reported for the OES using the 4-week recall period originally assigned to the instrument.

Construct validity, assessed by correlating scores for PROMs at T3 (3-5 months from injury), was good. The magnitude of the coefficient (*r*_s_) for the correlation between the OES total and QuickDASH scores (−0.91) surpassed the a priori hypothesis (≤−0.75). Likewise, Spearman’s rho surpassed the a priori hypothesis (≤−0.60) for correlations between scores for the domains of the OES and the QuickDASH. Most previous studies have assessed the construct validity of the OES by correlating its scores with those of the DASH^8^^,^^9^^,^^33^ or QuickDASH.^16^^,^^18^^,^^27^^,^^29^ Compared with these studies, all of which have applied a 4-week recall period for the OES, the correlation in the current study was comparable^8^^,^^27^^,^^29^ or stronger.^9^^,^^16^^,^^18^^,^^33^ The QuickDASH was used in the current study, but comparing our results with those of studies using the DASH also appears reasonable, as these two scores have shown similar behavior in terms of both construct validity and responsiveness.^22^^,^^47^ Responsiveness for improvement of the OES was assessed by correlating change scores (T3 minus T2) of the OES and QuickDASH, with the results indicating favorable characteristics. Correlation coefficients surpassed the a priori hypothesis of a magnitude of at least 0.5 for both the OES total score and its domains. Responsiveness for improvement appears similar to or better than in previous studies using a 4-week recall period and equivalent methodology for assessing responsiveness.^7^^,^^18^ To the best of our knowledge, responsiveness for deterioration has not been previously assessed for the OES. The correlation between OES total and QuickDASH change scores (T2 minus T1) was high (*r*_s_ = −0.88), indicating good responsiveness for deterioration.

The construct validity and responsiveness of the OES were also assessed by correlations with SANE-F. This analysis supported good construct validity, but correlations between SANE-F and OES change scores were low to moderate, indicating less than ideal responsiveness. The correlations between SANE-F and QuickDASH change scores were, however, also unfavorable while the correlation between OES and QuickDASH changes scores were high, suggesting a problem with the responsiveness of SANE-F. The responsiveness of the SANE in any form has, to the best of our knowledge, not previously been assessed for elbow conditions. A potential selective reduction in the construct validity of SANE-F for greater degrees of impairment might contribute to these findings. A reduction of this kind is indicated by previous studies^2^^,^^38^^,^^46^ and the low correlation between SANE-F values and OES scores at T2, a time point when function was notably impaired. Poor construct validity of SANE-F at T2 would be expected to affect responsiveness negatively, as T2 values are included in calculations for assessing responsiveness.

The OES showed good reliability. Internal consistency displayed good characteristics with Cronbach’s alpha values for the domains of the OES between 0.83 and 0.91, which falls within the range of what has previously been defined as adequate (0.70-0.95).^43^ Intraclass correlation coefficients were calculated to assess intrarater reliability, which resulted in excellent values (greater than 0.9) for the function and social-psychological domains and a value of 0.89 for the pain domain, which is considered to be good.^20^

The favorable measurement properties observed for the OES in this study, in particular for responsiveness, may be attributable to some extent to the use of a shortened 7-day recall period. One potential advantage of shorter recall periods is greater accuracy in recollection, which would be expected to have a positive effect on measurement properties. This idea has been embraced by the Food and Drug Administration, which prefers the use of short recall periods for PROs used to support claims in approved medical product labeling.^48^ An advantage of shorter recall periods was demonstrated in a study of patients with hip and knee osteoarthritis, which found that a 7-day recall period for pain ratings had a higher correlation with momentary ratings than a 4-week recall period.^31^ Greater accuracy in recollection might be a contributing factor to the improvement in responsiveness observed for some PROMs when used with shorter recall periods,^6^^,^^19^ exemplified by the 36-item short form survey (1 week vs. 4 weeks). Another potentially beneficial attribute of using a 7-day recall period for the OES is that correlations with the QuickDASH, which also has a 7-day recall period, might be more favorable than when a 4-week recall period is used, as responses would be expected to be more coherent when based on symptoms experienced during the same period of time.

Using a recall period shorter than the 4 weeks initially assigned to the OES might also be beneficial when a systematic increase or decrease (temporal trends) in symptoms can be expected to occur over 4 weeks, for example, while patients are recovering from an injury or surgery, as was the case for the patients included in the current study. In fact, the presence of variations in symptoms over the course of a recall period,^5^^,^^11^^,^^41^ in particular temporal trends,^40^ has been found to threaten the accuracy of rating symptoms by recall. It appears reasonable that longer recall periods might be more vulnerable to these effects. In the face of a variation in symptoms over the course of a recall period, patients might have difficulty summarizing their experiences when responding to items. Individuals might use different strategies to address such situations which would be expected to result in systematic variation and compromised validity. As a result, using a shortened recall period (eg, 7 days) for the OES might be preferable when making assessments during periods when temporal trends in symptoms are to be expected. Meanwhile, considering the model suggested by Stull et al^42^ for selecting the length of the recall period for PROMs, a 7-day recall period would appear reasonable for assessing elbow function in other situations as well, including assessments of patients in a more stable state.

The OES appears to be suitable for the assessment of the outcome of patients with elbow trauma as indicated by the favorable measurement properties observed in the current study which included exclusively patients with elbow injuries, predominantly fractures and dislocations. The results of two previous studies give some support to using the OES for assessing patients with elbow trauma. Padovani et al^29^ reported favorable reliability (internal consistency) and construct validity based on a group of 110 patients whereof 77 (70%) had been treated surgically for an elbow injury. Based on an analysis of 99 patients, Iordens et al^18^ concluded that the OES was a reliable (internal consistency), valid, and responsive instrument for assessing elbow function following nonsurgical treatment of simple elbow dislocation. Taken together, available literature indicates that the OES is suitable for the assessment of the outcome of both surgically and nonsurgically treated elbow injuries.

### Strengths and limitations

This study has some strengths. To the best of our knowledge, the measurement properties of the OES using a shortened recall period have not previously been described. Moreover, previous studies correlating OES and QuickDASH scores have generally not attempted to minimize the effect of upper extremity symptoms other than those from the elbow, as was done in the present study. These symptoms may be detected by the QuickDASH, obscuring correlations with the OES for elbow conditions specifically. As a result, patients with concurrent injuries or pre-existing conditions affecting the upper extremities were not included. The latter was defined as >10 points on the preinjury QuickDASH (T1). This cutoff appeared reasonable based on population-based normative data^1^ and estimates of measurement error.^12^^,^^18^ Finally, the responsiveness of the OES for deterioration was found to be good, a parameter which has rarely been reported in previous literature. One limitation is that the size of the group for the primary analysis of responsiveness (n = 35) did not meet the minimum of 50 patients that is regarded as adequate, according to the COSMIN checklist.^23^ A secondary analysis was therefore conducted by adding patients who responded to T2 between 4 and 6 days from injury based on their experiences so far during the first postinjury week (n = 57). The correlation coefficients remained similar to those from the primary analysis and above 0.8 for correlations between OES total and QuickDASH change scores for both improvement and deterioration.

## Conclusions

In this study, the OES demonstrated good measurement properties when used with a shortened 7-day recall period. These results further establish the OES as a well-validated, elbow-specific PROM and support its use with a shortened 7-day recall period.

## Acknowledgments

The authors thank Albin Jorméus at Sahlgrenska University Hospital and the orthopedic surgeons working at the upper extremity unit at Linköping University Hospital, who helped with data collection. The authors also thank Terez Zara for help with administrative work and The Gothenburg Society of Medicine for funding.

## Disclaimers

Funding: Eythor Örn Jonsson received a grant from The Gothenburg Society of Medicine, reference number GLS-961297. The outside source of funds was not involved in data collection, data analysis, or the preparation of or editing of the manuscript.

Conflicts of interest: No authors, their immediate families, and any research foundation with which they are affiliated have received any financial payments or other benefits from any commercial entity related to the subject of this article.
